# Applications of Spectral Gradient Algorithm for Solving Matrix *ℓ*_2,1_-Norm Minimization Problems in Machine Learning

**DOI:** 10.1371/journal.pone.0166169

**Published:** 2016-11-18

**Authors:** Yunhai Xiao, Qiuyu Wang, Lihong Liu

**Affiliations:** 1 Institute of Applied Mathematics, School of Mathematics and Statistics, Henan University, Kaifeng, Henan Province, China; 2 School of Mathematics and Statistics, Henan University, Kaifeng, Henan Province, China; 3 School of Mathematics and Statistics, Henan University, Kaifeng, Henan Province, China; Beihang University, CHINA

## Abstract

The main purpose of this study is to propose, then analyze, and later test a spectral gradient algorithm for solving a convex minimization problem. The considered problem covers the matrix *ℓ*_2,1_-norm regularized least squares which is widely used in multi-task learning for capturing the joint feature among each task. To solve the problem, we firstly minimize a quadratic approximated model of the objective function to derive a search direction at current iteration. We show that this direction descends automatically and reduces to the original spectral gradient direction if the regularized term is removed. Secondly, we incorporate a nonmonotone line search along this direction to improve the algorithm’s numerical performance. Furthermore, we show that the proposed algorithm converges to a critical point under some mild conditions. The attractive feature of the proposed algorithm is that it is easily performable and only requires the gradient of the smooth function and the objective function’s values at each and every step. Finally, we operate some experiments on synthetic data, which verifies that the proposed algorithm works quite well and performs better than the compared ones.

## 1 Introduction

The tasks in medical diagnosis [1], text classification [2–5], biomedical informatics [6, 7] and other applications [8–12] are always related to each other. Hence, capturing the shared information among each task becomes the key issue to learn [13–15]. Given the training set of *t* tasks $A=\left[A_{1};\ldots ;A_{t}\right]\in R^{m\times n}$ and $b={\left[b_{1};\ldots ;b_{t}\right]}^{⊤}\in R^{m}$, where *A*_*j*_ is the data for the *j*-th task and *b*_*j*_ is the corresponding response. We let $x_{j}\in R^{n}$ be the sparse feature for the *j*-th task, and let $X=\left[x_{1},\ldots ,x_{t}\right]\in R^{n\times t}$ be the joint feature to be learned. In order to select features globally, it encourages several rows of *X* to be zeros and solves the following *ℓ*_2,1_-norm regularized least squares [16, 17]

$$\begin{array}{c} \min_{X\in R^{n\times t}}\;\frac{1}{2}{∥AX-b∥}_{2}^{2}+\mu {∥X∥}_{2,1}, \end{array}$$ (1)

where *μ* > 0 is a weighting parameter, and ‖*X*‖_2,1_ is defined by the sum of the *ℓ*_2_-norm of each row of a matrix. It is well known that the *ℓ*_2,1_-norm is used to encourage the multiple predictions from different tasks to share similar parameter sparsity patterns.

In the past few years, several algorithms have been proposed, analyzed, and tested to solve the nonsmooth convex minimization Problem (1). The algorithm in [18] transformed Eq (1) equivalently into a smooth convex optimization problem and minimized consequently by Nesterov’s gradient method. The method in [16] reformulated Eq (1) as a constrained optimization problem and minimized alternately. The algorithm in [19] and its variant [20] reformulated the problem as an equivalent constrained minimization by introducing an auxiliary variable, and then minimized the corresponding augmented Lagrange function alternatively. Finally, for another accelerated proximal gradient version of the algorithm [19], one can refer to [21].

Unlike all the research activities which mainly concerned about Problem (1), in this paper, we focus on the following generalized nonsmooth convex optimization problem

$$\begin{array}{c} \min_{X\in R^{n\times t}}\;F\left(X\right)+\mu {∥X∥}_{2,1}, \end{array}$$ (2)

where $F:R^{n\times t}\to R$ is continuously differentiable (may be non-convex) and bounded below. Clearly, Model (2) includes Eq (1) as a special case when *F* is a least square. As we all know, the spectral gradient method was originated by Barzilai and Borwein [22] for solving smooth unconstrained minimization problems, later was developed in [23–26], and then was extended to solve *ℓ*_1_-regularized nonsmooth minimization [27]. However, its numerical performance in solving matrix *ℓ*_2,1_-norm involved nonsmooth minimization problems is still undiscovered. Therefore, extending the spectral gradient algorithm to solve Problem (2) may have significance both in theory and practice. The first contribution of this study lies in the design of the search direction at each iteration, which is derived by minimizing a quadratic approximated model of the objective function and at the same time making full use of the special structure of the *ℓ*_2,1_-norm. We also show that the generated direction descends automatically provided that the spectral coefficient is positive. The second contribution of the paper is the nonmonotone line search, which is used to improve the algorithm’s performance. At each iteration, the algorithm requires the gradient of the smooth term and the value of the objective function, which means it has the ability to solve high dimensional problems. Finally, we do performance comparisons with a couple of solvers IAMD_MFL and SLEP, which illustrate that the proposed method is fast, efficient, and competitive.

The paper is organized as follows. In Section 2, we provide some notations and preliminaries, and construct the new algorithm together with its properties. In Section 3, we establish the global convergence of the algorithm. In Section 4, we report some numerical results and do some performance comparisons. Finally, we conclude our paper in Section 5.

## 2 Algorithm

### 2.1 Notations and preliminaries

In the first place, we summarize the notations used in this paper. Matrices are written as uppercase letters. Vectors are described as lowercase letters. For the matrix *X*, its *i*-th row and *j*-th column are denoted by *X*_*i*,:_ and *X*_:,*j*_ respectively. The Frobenius norm and the *ℓ*_2,1_-norm of the matrix $X\in R^{n\times t}$ are defined as, respectively,

$$\begin{array}{c} {∥X∥}_{F}=\sqrt{\sum_{i=1}^{n}\sum_{j=1}^{t}X_{i,j}^{2}},\;\text{and}\;{∥X∥}_{2,1}=\sum_{i=1}^{n}\sqrt{\sum_{j=1}^{t}X_{i,j}^{2}}=\sum_{i=1}^{n}{∥X_{i,:}∥}_{2}. \end{array}$$

For any two matrices $X,Y\in R^{n\times t}$, we define 〈*X*, *Y*〉 = tr(*X*^⊤^ *Y*) (the standard trace inner product in $R^{t}$), so that ${∥X∥}_{F}=\sqrt{\langle X,X\rangle }$. If $x\in R^{d}$, we denote “Diag(*x*)” the diagonal matrix possessing the components of vector *x* on the diagonal. We define “⊤” as the transpose of a vector or a matrix. For the sake of simplicity, we let Φ(*X*) = *F*(*X*) + *μ*‖*X*‖_2,1_. Additional notations will be introduced when they occur.

We now quickly review the spectral gradient method for the unconstrained smooth minimization problem

$$\begin{array}{c} \min f\left(x\right),\;\;x\in R^{n}, \end{array}$$

where $f:R^{n}\to R$ is a continuously differentiable function. The spectral gradient method is defined by

$$\begin{array}{c} x_{k+1}=x_{k}-\lambda_{k}^{-1}\nabla f\left(x_{k}\right), \end{array}$$

where one of the choices of *λ*_*k*_ (named as spectral coefficient) is given by

$$\begin{array}{c} \lambda_{k}=\frac{s_{k-1}^{⊤}y_{k-1}}{{∥s_{k-1}∥}_{2}^{2}}, \end{array}$$

where *s*_*k*−1_ = *x*_*k*_ − *x*_*k*−1_ and *y*_*k*−1_ = ∇*f*(*x*_*k*_) − ∇*f*(*x*_*k*−1_). Obviously, if $s_{k-1}^{⊤}y_{k-1}>0$, i.e. *λ*_*k*_ > 0, the search direction $d_{k}:=-\lambda_{k}^{-1}\nabla f\left(x_{k}\right)$ descends automatically at current point.

### 2.2 Algorithm

Now, we turn our attention to the original Model (2). Since the *ℓ*_2,1_-norm is nodifferentiable, we approximate the objective function by the following quadratic function *Q*_*k*_:

$$\begin{array}{ccc} Q_{k}\left(D\right): & = & F\left(X_{k}+D\right)+\mu {∥X_{k}+D∥}_{2,1}\\ & \approx & F\left(X_{k}\right)+\left\langle \nabla F\left(X_{k}\right),D\right\rangle +\frac{\Lambda_{k}}{2}{∥D∥}_{F}^{2}+\mu {∥X_{k}+D∥}_{2,1}, \end{array}$$ (3)

where $\nabla F\left(X_{k}\right)\in R^{n\times t}$ is the gradient of *F* at *X*_*k*_; Λ_*k*_ is the so-called spectral coefficient which defined by

$$\begin{array}{c} \Lambda_{k}=\frac{\left\langle S_{k-1},Y_{k-1}\right\rangle }{{∥S_{k-1}∥}_{F}^{2}}, \end{array}$$ (4)

where *S*_*k*−1_ = *X*_*k*_ − *X*_*k*−1_ and *Y*_*k*−1_ = ∇*F*(*X*_*k*_) − ∇*F*(*X*_*k*−1_). Minimizing Eq (3) yields

$$\begin{array}{ccc} & & \text{arg}\min_{D\in R^{n\times t}}Q_{k}\left(D\right)\\ & & \;=\text{arg}\min_{D\in R^{n\times t}}\left\langle \nabla F\left(X_{k}\right),D\right\rangle +\frac{\Lambda_{k}}{2}{∥D∥}_{F}^{2}+\mu {∥X_{k}+D∥}_{2,1}\\ & & \;=\text{arg}\min_{D\in R^{n\times t}}\frac{1}{\Lambda_{k}}(\left\langle \nabla F\left(X_{k}\right),D\right\rangle +\frac{\Lambda_{k}}{2}{∥D∥}_{F}^{2}+\mu {∥X_{k}+D∥}_{2,1})\\ & & \;=\text{arg}\min_{D\in R^{n\times t}}\frac{1}{2}{∥X_{k}+D-(X_{k}-\frac{1}{\Lambda_{k}}\nabla F\left(X_{k}\right))∥}_{F}^{2}+\frac{\mu }{\Lambda_{k}}{∥X_{k}+D∥}_{2,1}. \end{array}$$

Denote *M*_*k*_ = *X*_*k*_ + *D* and $N_{k}=X_{k}-\frac{1}{\Lambda_{k}}\nabla f\left(X_{k}\right)$. One can get

$$\begin{array}{c} \text{arg}\min_{D\in R^{n\times t}}Q_{k}\left(D\right)=\text{arg}\min_{D\in R^{n\times t}}\sum_{i=1}^{n}(\frac{1}{2}{∥{\left(M_{k}\right)}_{i,:}-{\left(N_{k}\right)}_{i,:}∥}_{2}^{2}+\frac{\mu }{\Lambda_{k}}{∥{\left(M_{k}\right)}_{i,:}∥}_{2}). \end{array}$$ (5)

The favorable structure of Eq (5) make the *i*-th row of matrix *M*_*k*_ write explicitly as

$$\begin{array}{c} {\left(M_{k}\right)}_{i,:}=\max\{{∥{\left(N_{k}\right)}_{i:,}∥}_{2}-\frac{\mu }{\Lambda_{k}},0\}\frac{{\left(N_{k}\right)}_{i,:}}{{∥{\left(N_{k}\right)}_{i,:}∥}_{2}}, \end{array}$$

where the convention 0 ⋅ 0/0 = 0 is followed. Hence, the search direction at current point can be expressed as

$$\begin{array}{ccc} {\left(D_{k}\right)}_{i,:} & = & -[{\left(X_{k}\right)}_{i,:}-{\left(M_{k}\right)}_{i,:}]\\ & = & -[{\left(X_{k}\right)}_{i,:}-\max\{{∥{\left(N_{k}\right)}_{i:,}∥}_{2}-\frac{\mu }{\Lambda_{k}},0\}\frac{{\left(N_{k}\right)}_{i,:}}{{∥{\left(N_{k}\right)}_{i,:}∥}_{2}}]\\ & = & -[{\left(X_{k}\right)}_{i,:}-\max\{{∥{\left(X_{k}-\frac{1}{\Lambda_{k}}\nabla F\left(X_{k}\right)\right)}_{i:,}∥}_{2}-\frac{\mu }{\Lambda_{k}},0\}\frac{{\left(X_{k}-\frac{1}{\Lambda_{k}}\nabla F\left(X_{k}\right)\right)}_{i,:}}{{∥{\left(X_{k}-\frac{1}{\Lambda_{k}}\nabla F\left(X_{k}\right)\right)}_{i,:}∥}_{2}}]. \end{array}$$ (6)

Obviously, the Eq (6) reduces to $D_{k}=-\Lambda_{k}^{-1}\nabla F\left(x_{k}\right)$ at the case of *μ* = 0, which means Eq (6) covers the traditional spectral gradient direction as a special case.

The following lemma verifies that *D*_*k*_ is a descent direction when the optimal solution is not achieved.

**Theorem 1** Suppose that Λ_*k*_ > 0 and *D*_*k*_ is determined by Eq (6). Then

$$\begin{array}{c} \Phi \left(X_{k}+\theta D_{k}\right)\leq \Phi \left(X_{k}\right)+\theta [\left\langle \nabla F\left(X_{k}\right),D_{k}\right\rangle +\mu {∥X_{k}+D_{k}∥}_{2,1}-\mu {∥X_{k}∥}_{2,1}]+o\left(\theta \right),\;\theta \in \left(0,1\right], \end{array}$$ (7)

and

$$\begin{array}{c} \left\langle \nabla F\left(X_{k}\right),D_{k}\right\rangle +\mu {∥X_{k}+D_{k}∥}_{2,1}-\mu {∥X_{k}∥}_{2,1}\leq -\frac{\Lambda_{k}}{2}{∥D_{k}∥}_{F}^{2}. \end{array}$$ (8)

**Proof.** By the differentiability of *F* and the convexity of ‖*X*‖_2,1_, we have that for any *θ* ∈ (0, 1],

$$\begin{array}{ccc} & & \Phi \left(X_{k}+\theta D_{k}\right)-\Phi \left(X_{k}\right)\\ & & \;=F\left(X_{k}+\theta D_{k}\right)+\mu {∥X_{k}+\theta D_{k}∥}_{2,1}-F\left(X_{k}\right)-\mu {∥X_{k}∥}_{2,1}\\ & & \;=F\left(X_{k}+\theta D_{k}\right)-F\left(X_{k}\right)+\mu {∥\theta \left(X_{k}+D_{k}\right)+\left(1-\theta \right)X_{k}∥}_{2,1}-\mu {∥X_{k}∥}_{2,1}\\ & & \;\leq F\left(X_{k}+\theta D_{k}\right)-F\left(X_{k}\right)+\theta \mu {∥X_{k}+D_{k}∥}_{2,1}+\left(1-\theta \right)\mu {∥X_{k}∥}_{2,1}-\mu {∥X_{k}∥}_{2,1}\\ & & \;=\theta \left\langle \nabla F\left(X_{k}\right),D_{k}\right\rangle +o\left(\theta \right)+\theta [\mu {∥X_{k}+D_{k}∥}_{2,1}-\mu {∥X_{k}∥}_{2,1}], \end{array}$$

which is exactly Eq (7). Noting that *D*_*k*_ is the minimizer of Eq (3) and *θ* ∈ (0, 1], by Eq (3) and the convexity of ‖*X*‖_2,1_, one can get

$$\begin{array}{ccc} & & \left\langle \nabla F\left(X_{k}\right),D_{k}\right\rangle +\frac{\Lambda_{k}}{2}{∥D_{k}∥}_{F}^{2}+\mu {∥X_{k}+D_{k}∥}_{2,1}-\mu {∥X_{k}∥}_{2,1}\\ & & \;\leq \left\langle \nabla F\left(X_{k}\right),\theta D_{k}\right\rangle +\frac{\Lambda_{k}}{2}{∥\theta D_{k}∥}_{F}^{2}+\mu {∥X_{k}+\theta D_{k}∥}_{2,1}-\mu {∥X_{k}∥}_{2,1}\\ & & \;\leq \left\langle \nabla F\left(X_{k}\right),\theta D_{k}\right\rangle +\frac{\Lambda_{k}\theta^{2}}{2}{∥D_{k}∥}_{F}^{2}+\theta \mu {∥X_{k}+D_{k}∥}_{2,1}+\mu \left(1-\theta \right){∥X_{k}∥}_{2,1}-\mu {∥X_{k}∥}_{2,1}. \end{array}$$

Hence,

$$\begin{array}{c} \left(1-\theta \right)\left\langle \nabla F\left(X_{k}\right),D_{k}\right\rangle +\mu \left(1-\theta \right){∥X_{k}+D_{k}∥}_{2,1}-\mu \left(1-\theta \right){∥X_{k}∥}_{2,1}\leq -\frac{\Lambda_{k}}{2}\left(1-\theta^{2}\right){∥D_{k}∥}_{F}^{2}, \end{array}$$

i.e.,

$$\begin{array}{c} \left\langle \nabla F\left(X_{k}\right),D_{k}\right\rangle +\mu {∥X_{k}+D_{k}∥}_{2,1}-\mu {∥X_{k}∥}_{2,1}\leq -\frac{\Lambda_{k}}{2}\left(1+\theta \right){∥D_{k}∥}_{F}^{2}. \end{array}$$

Recalling *θ* ∈ (0, 1], the above inequality indicates Eq (8) is correct. ♯

To improve the algorithm’s performance, we use the classical nonmonotone line search [28] to find a suitable stepsize along the direction. It is well known that this technique allows the functional values to increase occasionally in some iterations but decrease in the whole iterative process. Letting *δ* ∈ (0, 1), *ρ* ∈ (0, 1) and $\tilde{m}$ be a given positive integer, we choose the smallest nonnegative integer *j*_*k*_ such that the stepsize $\alpha_{k}=\tilde{\alpha }\rho^{j_{k}}$ satisfies

$$\begin{array}{c} \Phi \left(X_{k}+\alpha_{k}D_{k}\right)\leq \max_{0\leq j\leq m(k)}\Phi \left(X_{k-j}\right)+\delta \alpha_{k}\Delta_{k}, \end{array}$$ (9)

where $0\leq m\left(k\right)\leq \min\{m\left(k-1\right)+1,\tilde{m}\}$ (*m*(0) = 0) and

$$\begin{array}{c} \Delta_{k}=\left\langle \nabla F\left(X_{k}\right),D_{k}\right\rangle +\mu {∥X_{k}+D_{k}∥}_{2,1}-\mu {∥X_{k}∥}_{2,1}. \end{array}$$ (10)

From Eq (8), it is clear that $\Delta_{k}\leq -\frac{\Lambda_{k}}{2}{∥D_{k}∥}_{F}^{2}<0$ whenever *D*_*k*_ ≠ 0, which shows that Eq (9) is well-defined.

In summary, the full steps of the **N**onmonotone **S**pectral **G**radient algorithm for ***L*_2,1_**-norm minimization (abbr. NSGL21) can be described as follows:

**Algorithm 1 (NSGL21)**

**Step 0.** Choose initial point *X*_0_, constants *μ* > 0, $\tilde{\alpha }>0$, *ρ* ∈ (0, 1), *δ* ∈ (0, 1) and positive integer $\tilde{m}$. Set *k*: = 0.

**Step 1.** Stop if ‖*D*_*k*_‖_*F*_ = 0. Otherwise, continue.

**Step 2.** Compute *D*_*k*_ via Eq (6).

**Step 3.** Compute *α*_*k*_ via Eq (9).

**Step 4.** Let *X*_*k*+1_: = *X*_*k*_+*α*_*k*_ *d*_*k*_.

**Step 5.** Let *k*: = *k*+1. Go to Step 1.

As is stated in the proceeding section that the generated direction descend automatically whenever Λ_*k*_ > 0. To ensure Λ_*k*_ > 0, we choose a sufficiently small Λ_(min)_ > 0 and a sufficiently large Λ_(max)_ > 0, such that Λ_*k*_ is forced as

$$\begin{array}{c} \Lambda_{k}:=\min\left\{\Lambda_{\left(\max\right)},\max\left\{\Lambda_{k},\Lambda_{\left(\min\right)}\right\}\right\}. \end{array}$$

This approach ensures that the hereditary descent property is guaranteed at each and every step.

**Remark 1.** The steps of the proposed algorithm is novel and different to other existing approaches. The well-known approach [18] reformulated Problem (2) as the following constrained smooth convex optimization problem

$$\begin{array}{c} \min_{X\in R^{n\times t},\xi \in R^{n}}\;\{F\left(X\right)+\mu \sum_{i=1}^{n}\xi_{i}\;|\;∥X_{i,:}∥\leq \xi_{i}\}, \end{array}$$

and then solved via the Nesterov’s method. The method in [19] paid attention least square Model (1) and used an auxiliary variable to transform the model equivalently as

$$\begin{array}{c} \min_{X\in R^{n\times t}}\{\frac{1}{2}{∥Y∥}_{2}^{2}+\mu {∥X∥}_{2,1}\;|\;AX-b=Y\}. \end{array}$$

An alternating direction method of multiplier is used immediately to solve the resulting model and closed-form solution are derived at each subproblem. Clearly, our proposed algorithm is different from the above mentioned approaches in sense that we solve the original Model (2) directly without any transformation. ♯

## 3 Convergence analysis

This section is devoted to establishing the global convergence of algorithm NSGL21. For this purpose, we make the following assumption.

**Assumption 1.** The level set *Ω* = {*X*: *F*(*X*) ≤ *F*(*X*_0_)} is bounded.

**Lemma 2.** Suppose that the Assumption 1 holds and the sequence {*X*_*k*_} is generated by Algorithm 1. Then *X*_*k*_ is a stationary point of Problem (2) if and only if *D*_*k*_ = 0.

**Proof.** In the case of *D*_*k*_ ≠ 0, Lemma 1 shows that *D*_*k*_ is a descent direction, which implies that *X*_*k*_ is not a stationary point of *F*. On the other hand, since *D*_*k*_ = 0 is the solution of Eq (5), for any $\xi D\in R^{n\times t}$ with *ξ* > 0 we have

$$\begin{array}{c} \left\langle \nabla F\left(X_{k}\right),\xi D\right\rangle +\frac{\Lambda_{k}\xi^{2}}{2}{∥D∥}_{F}^{2}+\mu {∥X_{k}+\xi D∥}_{2,1}\geq \mu {∥X_{k}∥}_{2,1}. \end{array}$$ (11)

Combining the fact *F*(*X*_*k*_ + *ξD*) − *F*(*X*_*k*_) = 〈∇*F*(*X*_*k*_), *ξD*〉 + *o*(*ξ*) with Eq (11), it yields

$$\begin{array}{ccc} \Phi^{\prime }\left(X_{k};D\right) & = & \lim_{\xi ↓0}\frac{F\left(X_{k}+\xi D\right)-F\left(X_{k}\right)+\mu {∥X_{k}+\xi D∥}_{2,1}-\mu {∥X_{k}∥}_{2,1}}{\xi }\\ & = & \lim_{\xi ↓0}\frac{\xi \left\langle \nabla F\left(X_{k}\right),D\right\rangle +o\left(\xi \right)+\mu {∥X_{k}+\xi D∥}_{2,1}-\mu {∥X_{k}∥}_{2,1}}{\xi }\\ & \geq & \lim_{\xi ↓0}\frac{-\frac{\Lambda_{k}\xi^{2}}{2}{∥D∥}_{F}^{2}+o\left(\xi \right)}{\xi }\\ & = & 0, \end{array}$$

which indicates that *X*_*k*_ is a stationary point of *F*. ♯

**Lemma 3.** Let *l*(*k*) be an integer such that

$$\begin{array}{c} k-m\left(k\right)\leq l\left(k\right)\leq k\;\text{and}\;\Phi \left(X_{l(k)}\right)=\max_{0\leq j\leq m(k)}\Phi \left(X_{k-j}\right). \end{array}$$

Then the sequence {Φ(*X*_*l*(*k*)_)} is nonincreasing and the search direction *D*_*l*(*k*)_ satisfies

$$\begin{array}{c} \lim_{k\to \infty }\alpha_{l(k)}{∥D_{l(k)}∥}_{F}^{2}=0. \end{array}$$ (12)

**Proof.** It is not difficult to see that Φ(*X*_*l*(*k*+1)_) ≤ Φ(*X*_*l*(*k*)_), which indicates that the maximum value of the objective function is nonincreasing at each iteration. Moreover, by Eq (9), we have that for all $k>\tilde{m}$,

$$\begin{array}{ccc} \Phi (X_{l(k)}) & = & \Phi (X_{l(k)-1}+\alpha_{l(k)-1}D_{l(k)-1})\\ & \leq & \max_{0\leq j\leq m(l(k)-1)}\Phi \left(X_{l(k)-1-j}\right)+\delta \alpha_{l(k)-1}\Delta_{l(k)-1}\\ & = & \Phi \left(X_{l(l(k)-1)}\right)+\delta \alpha_{l(k)-1}\Delta_{l(k)-1}. \end{array}$$

By Assumption 1, the sequence {Φ(*X*_*l*(*k*)_)} admits a limit as *k* → ∞. Hence, it follows that

$$\begin{array}{c} \lim_{k\to \infty }\alpha_{l(k)}\Delta_{l(k)}=0. \end{array}$$ (13)

On the other hand, by the definition of Δ_*k*_ in Eq (10) and the inequality Eq (8), it is easy to deduce that

$$\begin{array}{c} \Delta_{l(k)}\leq -\frac{\Lambda_{\left(\min\right)}}{2}{∥D_{l(k)}∥}_{F}^{2}<0. \end{array}$$

Combining with Eq (13), one get

$$\begin{array}{c} \lim_{k\to \infty }\alpha_{l(k)}{∥D_{l(k)}∥}_{F}^{2}=0, \end{array}$$

which indicates the desirable result Eq (12). ♯

**Theorem 1.** Let the sequence {*X*_*k*_} and {*D*_*k*_} be generated by Algorithm 1. Then, there exists a subsequence k∈K such that

$$\begin{array}{c} \lim_{k\to \infty ,k\in K}{∥D_{k}∥}_{F}=0. \end{array}$$ (14)

**Proof.** Let $\bar{X}$ be a limit point of {*X*_*k*_}, and ${\left\{X_{k}\right\}}_{K_{1}}$ be a subsequence of {*X*_*k*_} converging to $\bar{X}$. Then by Eq (12) either $\left({∥\bar{D}∥}_{F}:=\right)\lim_{k\to \infty ,k\in K_{1}}{∥D_{k}∥}_{F}=0$, or there exists a subsequence ${\left\{X_{k}\right\}}_{K}$ ($K\subset K_{1}$) such that

$$\begin{array}{c} \lim_{k\to \infty ,k\in K}D_{k}\neq 0\;\text{and}\;\lim_{k\to \infty ,k\in K}\alpha_{k}=0. \end{array}$$ (15)

In this condition, we assume that there exists a constant *ϵ* > 0 such that

$$\begin{array}{c} {∥D_{k}∥}_{F}\geq \epsilon ,\;\forall \;k\in K. \end{array}$$ (16)

Since *α*_*k*_ is the first value to satisfy Eq (9), it follows from Step 3 in Algorithm 1 that there exists an index $\bar{k}$ such that, for all $k\geq \bar{k}$ and k∈K,

$$\begin{array}{c} \Phi \left(X_{k}+\frac{\alpha_{k}}{\rho }d_{k}\right)>\max_{0\leq j\leq m(k)}\Phi \left(X_{k-j}\right)+\delta \frac{\alpha_{k}}{\rho }\Delta_{k}\geq \Phi \left(X_{k}\right)+\delta \frac{\alpha_{k}}{\rho }\Delta_{k}. \end{array}$$ (17)

Since *F* is continuously differentiable, by the mean-value theorem on *F*, we can find that there exists a constant *θ*_*k*_ ∈ (0, 1), such that

$$\begin{array}{c} F\left(X_{k}+\frac{\alpha_{k}}{\rho }D_{k}\right)-F\left(X_{k}\right)=\frac{\alpha_{k}}{\rho }\left\langle \nabla F\left(X_{k}+\theta_{k}\frac{\alpha_{k}}{\rho }D_{k}\right),D_{k}\right\rangle . \end{array}$$

Combining with Eq (17), we have

$$\begin{array}{c} \left\langle \nabla F\left(X_{k}+\theta_{k}\frac{\alpha_{k}}{\rho }D_{k}\right),D_{k}\right\rangle +\frac{\mu {∥X_{k}+\frac{\alpha_{k}}{\rho }D_{k}∥}_{2,1}-\mu {∥X_{k}∥}_{2,1}}{\alpha_{k}/\rho }>\delta \Delta_{k}. \end{array}$$ (18)

Since *α*_*k*_ → 0 in Eq (15), we have *α*_*k*_ < *ρ* as *k* → ∞. It is not difficult to show that

$$\begin{array}{c} \frac{\mu {∥X_{k}+\frac{\alpha_{k}}{\rho }D_{k}∥}_{2,1}-\mu {∥X_{k}∥}_{2,1}}{\alpha_{k}/\rho }-[\mu {∥X_{k}+D_{k}∥}_{2,1}-\mu {∥X_{k}∥}_{2,1}]\leq 0. \end{array}$$ (19)

Subtracting left side of Eq (18) by Δ_*k*_ and noting the definition of Δ_*k*_, it is distinct that

$$\begin{array}{ccc} & & \left\langle \nabla f\left(X_{k}+\theta_{k}\frac{\alpha_{k}}{\rho }D_{k}\right),D_{k}\right\rangle +\frac{\mu {∥X_{k}+\frac{\alpha_{k}}{\rho }D_{k}∥}_{2,1}-\mu {∥X_{k}∥}_{2,1}}{\alpha_{k}/\rho }-\Delta_{k}\\ & & \;=\left\langle \nabla f\left(X_{k}+\theta_{k}\frac{\alpha_{k}}{\rho }D_{k}\right),D_{k}\right\rangle -\left\langle \nabla f\left(X_{k}\right),D_{k}\right\rangle \\ & & \;+[\frac{\mu {∥X_{k}+\frac{\alpha_{k}}{\rho }D_{k}∥}_{2,1}-\mu {∥X_{k}∥}_{2,1}}{\alpha_{k}/\rho }-(\mu {∥X_{k}+D_{k}∥}_{2,1}-\mu {∥X_{k}∥}_{2,1})]. \end{array}$$

Noting Eq (19), thus Eq (18) shows that

$$\begin{array}{ccc} & & \left\langle \nabla F\left(X_{k}+\theta_{k}\frac{\alpha_{k}}{\rho }D_{k}\right),D_{k}\right\rangle -\left\langle \nabla F\left(X_{k}\right),D_{k}\right\rangle \\ & & \;>-\left(1-\delta \right)\Delta_{k}\\ & & \;\geq \left(1-\delta \right)\frac{\Lambda_{\left(\min\right)}}{2}{∥D_{k}∥}_{F}^{2}. \end{array}$$ (20)

Taking the limit as k∈K, *k* → ∞ in the both sides of Eq (20) and using the smoothness of *F*, we obtain

$$\begin{array}{c} 0=\left\langle \nabla F\left(\bar{X}\right),\bar{D}\right\rangle -\left\langle \nabla F\left(\bar{X}\right),\bar{D}\right\rangle \geq \left(1-\delta \right)\frac{\Lambda_{\left(\min\right)}}{2}{∥\bar{D}∥}_{F}^{2}, \end{array}$$

which implies ‖*D*_*k*_‖_*F*_ → 0 as k∈K, *k* → ∞. This yields a contradiction because Eq (16) indicates that ‖*D*_*k*_‖_*F*_ is bounded. ♯

## 4 Numerical experiments

In this section, we present numerical results to illustrate the feasibility and efficiency of the algorithm NSGL21. In particular, we also test against the recent solvers IADM_MFL and SLEP for performance comparison. In running SLEP (Sparse Learning with Efficient Projections), we use the code at http://www.public.asu.edu/~jye02/Software/SLEP/index.htm in its Matlab package, and choose mFlag = 1 and lFlag = 1 for using an adaptive line search. All experiments are carried out under Windows 7 and Matlab v7.8 (2009a) running on a Lenovo laptop with an Intel Pentium CPU at 2.5 GHz and 4 GB of memory.

As [16], in the first test, ${\bar{X}}_{:,j}$ is generated from a 5-dimensional Gaussian distribution with zero-mean and con-variance diag{1, 0.64, 0.49, 0.36, 0.25}. Regarding each ${\bar{X}}_{:,j}$, we keep adding up to 20 irrelevant dimensions which are exactly zeros. The training and test data *A*_*j*_ is Gaussian matrices and their response data *b*_*j*_ is generated by

$$\begin{array}{c} b_{j}=A_{j}{\bar{X}}_{:,j}+\omega , \end{array}$$

where *ω* is zero-mean Gaussian noise with standard deviation 1.*e* − 2. We start NSGL21 from zero point and terminate the iterative process when

$$\begin{array}{c} {∥D_{k}∥}_{F}<tol, \end{array}$$ (21)

where *tol* > 0 is a tolerance. The quality of the solution X* is measured by the relative error to $\bar{X}$, i.e.,

$$\begin{array}{c} \text{RelErr}=\frac{{∥X^{*}-\bar{X}∥}_{F}}{{∥\bar{X}∥}_{F}}. \end{array}$$

**Fig 1:**
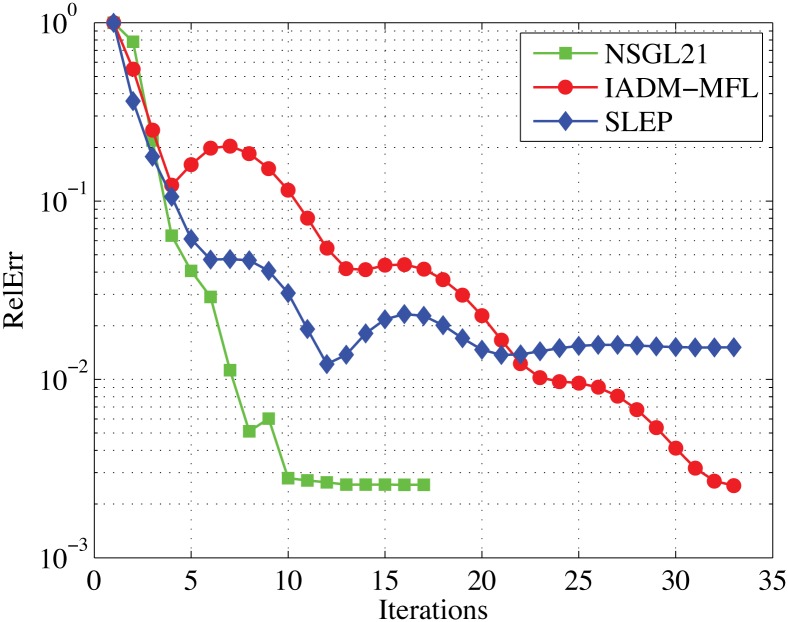
Comparison results of NSGL21, IADM MFL, and SLEP. The x-axes represents the number of iterations and the y-axes represents the relative error.

**Fig 2:**
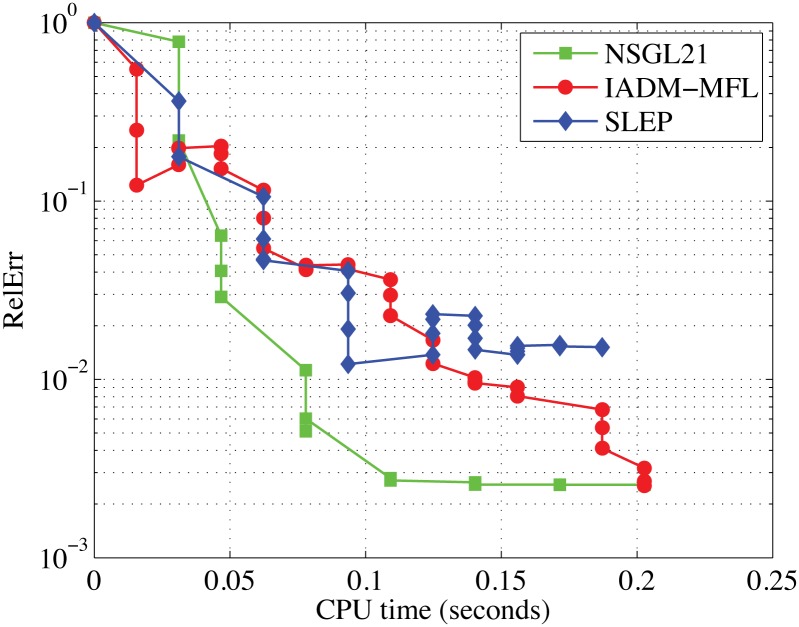
Comparison results of NSGL21, IADM MFL, and SLEP. The x-axes represents the CPU time in seconds and the y-axes represents the relative error.

In this test, we take $\tilde{\alpha }=1$, *μ* = 1*e* − 2, *t* = 200, *n* = 15, *tol* = 1*e* − 3, Λ_(min)_ = 10^−20^, Λ_(max)_ = 10^20^, and *m*_*j*_ = 100 for all *j* = 1, 2, …, *t*. Moreover, to compare the performance of these algorithms in a fair way, we run each code from zero point, use all the default parameter values, and observe their convergence behavior in obtaining similar accurate solutions. To specifically illustrate the performance of each algorithm, we draw a couple of figures to show their convergence behaviors with respect to the relative error and computing time proceed in Figs 1 and 2.

Observing Figs 1 and 2, we clearly know that IADM_MFL and NSGL21 produced faithful results expect for SLEP. We have tried to run SLEP with more iterations in our experiments’ preparation, but it cannot achieve progress any more. Meanwhile, NSGL21 requires less number of iterations than IADM_MFL to achieve the similar quality of solutions. In both plots, we see that the green line lies at the bottom of each plot in most cases, which indicates that NSGL21 is superior to the other two solvers.

The simple test is not enough to verify that NSGL21 is the winner. To further illustrate the benefit of NSGL21, we give some insights to the behavior of NSGL21 with different dimensions and different number of tasks. The results are listed in Table 1, which contains the number of iterations (Iter), the CPU time in seconds (Time), the relative errors (RelErr), and the final functional values (Fun).

**Table 1:** Comparison results of NSGL21 with IADM_MFL and SLEP.

		NSGL21				IADM_MFL				SLEP			
t	n	Iter	Time	Error	Fun	Iter	Time	Error	Fun	Iter	Time	Error	Fun
50	5	12	0.03	1.32e-3	0.49	23	0.05	3.49e-3	0.53	32	0.06	1.66e-2	2.27
50	10	12	0.03	1.95e-3	0.48	32	0.06	2.28e-3	0.49	29	0.06	1.67e-2	2.26
50	15	14	0.03	2.33e-3	0.47	34	0.08	2.53e-3	0.48	29	0.03	1.67e-2	2.26
50	20	15	0.03	3.00e-3	0.45	39	0.06	2.79e-3	0.46	30	0.06	1.66e-2	2.26
50	25	14	0.05	3.49e-3	0.44	42	0.06	2.87e-3	0.45	33	0.09	1.65e-2	2.25
100	5	11	0.06	1.39e-3	0.83	24	0.05	1.62e-3	0.84	32	0.09	1.51e-2	3.61
100	10	12	0.05	2.13e-3	0.81	29	0.06	2.25e-3	0.83	41	0.09	1.52e-2	3.66
100	15	17	0.06	2.49e-3	0.79	33	0.09	2.55e-3	0.82	32	0.12	1.49e-2	3.57
100	20	15	0.09	2.99e-3	0.75	38	0.11	2.37e-3	0.79	32	0.11	1.50e-2	3.59
100	25	19	0.12	3.43e-3	0.74	43	0.14	2.72e-3	0.80	28	0.16	1.55e-2	3.73
150	5	12	0.06	1.43e-3	1.14	24	0.08	1.81e-3	1.16	35	0.14	1.51e-2	5.19
150	10	14	0.09	1.98e-3	1.11	29	0.09	2.44e-3	1.15	33	0.16	1.49e-2	5.18
150	15	17	0.12	2.57e-3	1.08	34	0.17	2.91e-3	1.15	32	0.22	1.51e-2	5.20
150	20	15	0.16	3.04e-3	1.03	40	0.20	2.79e-3	1.11	35	0.17	1.50e-2	5.16
150	25	19	0.22	3.45e-3	0.99	45	0.23	3.00e-3	1.08	35	0.28	1.49e-2	5.14
200	5	12	0.12	1.41e-3	1.45	24	0.12	1.68e-3	1.46	45	0.12	1.53e-2	7.10
200	10	12	0.12	1.94e-3	1.41	29	0.19	2.09e-3	1.45	41	0.14	1.53e-2	7.10
200	15	17	0.19	2.57e-3	1.35	33	0.25	2.54e-3	1.41	33	0.25	1.51e-2	6.98
200	20	15	0.19	3.10e-3	1.32	38	0.25	3.09e-3	1.41	34	0.25	1.51e-2	6.95
200	25	19	0.28	3.52e-3	1.26	43	0.31	3.22e-3	1.35	27	0.28	1.57e-2	7.30
250	5	11	0.12	1.43e-3	1.74	24	0.17	1.58e-3	1.75	38	0.28	1.55e-2	8.80
250	10	14	0.25	2.01e-3	1.68	31	0.25	2.30e-3	1.74	37	0.31	1.55e-2	8.77
250	15	17	0.28	2.58e-3	1.61	36	0.28	3.00e-3	1.71	33	0.31	1.54e-2	8.70
250	20	15	0.31	3.02e-3	1.56	39	0.36	3.13e-3	1.66	34	0.37	1.53e-2	8.70
250	25	19	0.37	3.46e-3	1.50	46	0.45	3.63e-3	1.62	30	0.25	1.61e-2	9.26
300	5	12	0.22	1.40e-3	2.04	26	0.25	1.77e-3	2.07	35	0.28	1.54e-2	10.55
300	10	12	0.23	2.04e-3	1.96	30	0.27	2.31e-3	2.03	45	0.42	1.57e-2	10.77
300	15	17	0.37	2.52e-3	1.90	35	0.37	3.10e-3	2.03	35	0.39	1.53e-2	10.50
300	20	14	0.37	3.03e-3	1.83	41	0.51	3.52e-3	1.96	34	0.31	1.54e-2	10.52
300	25	20	0.58	3.52e-3	1.72	45	0.62	4.36e-3	1.91	29	0.44	1.62e-2	11.26

From Table 1, we clearly observe that each algorithm requires more computing time with the increase of the problems’ dimensions and the number of tasks. Meanwhile, the number of iterations required by NSGL21 and IADM_MFL increases slightly at the higher dimensions case. We also observe that, for all the tested problems, both NSGL21 and IADM_MFL are terminated abnormally in producing similar quality solutions in sense of comparable relative errors and final function values. However, SLEP cannot generate acceptable solutions although more iterations are permitted in experiments’ preparation. Hence, we conclude that NSGL21 and IADM_MFL perform better than SLEP. Now, we turn our attention to the performance comparison of solvers IADM_MFL and NSGL21. For getting similar quality of solutions, we take notice that NSGL21 is faster than IADM_MFL and saves at least 50% number of iterations. It is reasonable to make an conclusion that NSGL21 is the winner among the compared solvers.

## 5 Conclusions

In this paper, we have proposed, then analyzed, and later tested a nonmonotone spectral gradient algorithm for solving *ℓ*_2,1_-norm regularized minimization problem. The type of this problem mainly appears in computer version, text classification and biomedical informatics. Due to the nonsmoothness of the regularization term, the task of minimizing the problem is full of challenges. To the best of our knowledge, SLEP and IADM_MFL are the only available solvers of solving this problem. However, both solvers transferred equivalently to an equality-constrained minimization problem and then minimized alternatively. As we all know that the spectral gradient algorithm is very effective to solve smooth minimization problem. Hence, its performance in solving *ℓ*_2,1_-norm regularized problems is worthy of investigating. Certainly, it is the main motivation of our paper. At each iteration, the method proposed in this paper minimizes an approximal quadratic model of the objective function to produce a search direction. We showed that the generated direction descends automatically and the algorithm converges globally under some mild conditions. Additionally, the numerical experiments illustrate that the proposed algorithm is competitive with or even performs better than SLEP and IADM_MFL. Of course, this is the numerical contribution of our paper. We have said that the *ℓ*_2,1_-norm regularized minimization problem is partly arising in multi-task learning for capturing joint feather between each task. However, we did not test its real performance by using real data, this should be our further task to investigate. Finally, we expect that the proposed method and its extensions could produce even applications for problems in relevant areas of the machine learning.
